# Synergistic anti-tumor effect of combined inhibition of EGFR and JAK/STAT3 pathways in human ovarian cancer

**DOI:** 10.1186/s12943-015-0366-5

**Published:** 2015-05-01

**Authors:** Wei Wen, Jun Wu, Lucy Liu, Yan Tian, Ralf Buettner, Meng-Yin Hsieh, David Horne, Thanh H Dellinger, Ernest S Han, Richard Jove, John H Yim

**Affiliations:** Department of Molecular Medicine, Beckman Research Institute, City of Hope Comprehensive Cancer Center, 1500 East Duarte Rd., Duarte, CA 91010 USA; Department of Comparative Medicine, Beckman Research Institute, City of Hope Comprehensive Cancer Center, 1500 East Duarte Rd., Duarte, CA 91010 USA; Department of Surgery, Beckman Research Institute, City of Hope Comprehensive Cancer Center, 1500 East Duarte Rd., Duarte, CA 91010 USA; Vaccine & Gene Therapy Institute of Florida, 9801 SW Discovery Way, Port St. Lucie, 34987 Florida USA

**Keywords:** Ovarian cancer, EGFR resistance, JAK/STAT3, feedback, combination, synergy

## Abstract

**Background:**

The EGFR signaling pathway is frequently activated in human ovarian cancer and associated with poor prognosis. However, inhibition of EGFR signaling in patients with recurrent ovarian cancer has been disappointing. It remains to be addressed whether ovarian cancer patients could benefit from targeting EGFR signaling. Here we investigated the mechanisms underlying the resistance to EGFR inhibition in ovarian cancer and developed a strategy to overcome it.

**Results:**

We found that treatment of human ovarian cancer cells with an EGFR inhibitor, gefitinib, resulted in increased STAT3 phosphorylation in a dose- and time-dependent manner. Inhibiting STAT3 activation with a small molecule inhibitor of JAK, an upstream kinase that phosphorylates and activates STAT3, synergistically increased the anti-tumor activity of gefitinib *in vitro*. Similar results were obtained when STAT3 or JAK1 expression was knocked down. In contrast, inhibiting other signaling pathways, such as AKT/mTOR, MEK or SRC, was relatively less effective. The combined treatment resulted in simultaneous attenuation of multiple survival pathways and increased inhibition of ERK pathway. In addition, the dual inhibition showed a stronger suppression of xenograft tumor growth than either single inhibition.

**Conclusions:**

Our findings demonstrate that feedback activation of STAT3 pathway might contribute to the resistance to EGFR inhibition. Combined blockade of both pathways appears to be more effective against human ovarian cancer than inhibition of each pathway alone both *in vitro* and *in vivo*. This study may provide a strategy to improve clinical benefit of targeting EGFR pathway in ovarian cancer patients.

## Background

Ovarian cancer is the most lethal gynecologic malignancy in the Western world, with more than 21,980 new cases diagnosed and 14,270 deaths annually in the United States, according to the American Cancer Society. The 5-year survival rate for ovarian cancer patients ranges from 30-92%. Although the initial response rate to standard first line paclitaxel-platinum treatment is over 70%, most of the patients will eventually experience recurrence with a median progression-free survival of 18 months. Efforts have been made to overcome resistance using several classes of chemotherapy agents, however, mortality remains to be high in the platinum-resistant patients [1-3]. Therefore, there is a critical need to develop novel strategies that can be used to treat advanced and drug resistant ovarian cancer.

Substantial progress has been made in understanding the molecular events underlying ovarian cancer, which would facilitate the development of more effective targeted therapies [4-6]. A number of pro-survival signaling pathways is persistently activated in human ovarian cancer, including the epidermal growth factor receptor (EGFR) and janus kinase/signal transducer and activator of transcripton 3 (JAK/STAT3) pathways [4]. EGFR is a receptor tyrosine kinase that can be activated by extracellular ligands, leading to receptor autophosphorylation and subsequent activation of downstream pathways involved in proliferation, survival, angiogenesis, and invasion [7-9]. In cancer cells, the EGFR pathway is frequently activated [10-12]. Targeted inhibition of EGFR with a small molecule kinase inhibitor has been successful for lung cancer with EGFR mutations [13,14]. The EGF-receptor is over-expressed in 70% of ovarian cancers and associated with poor prognosis, suggesting that EGFR is an attractive therapeutic target in this cancer [12,15-19]. However, clinical trials with several different EGFR inhibitors have shown only modest activity. These trials have used gefitinib, either as a single agent or in combination with standard chemotherapy, in patients with recurrent disease [4,16,20-24]. Studies in non-small cell lung and other cancers have identified several resistance mechanisms [25-27]. However, little is known about the mechanism of resistance in ovarian cancer.

STAT3 is a member of the STAT family of transcription factors that mediate cellular responses to cytokines and growth factors. In normal tissue, STAT3 is latent and resides in the cytoplasm. In response to cytokine stimulation, STAT3 is phosphorylated at Tyr705 by JAK. Phosphorylated STAT3 can then translocate to the nucleus, bind to DNA and activate the transcription of various genes involved in cell survival and proliferation [28,29]. In contrast to normal cells, where activation of STAT3 is tightly regulated and transient, STAT3 is frequently constitutively activated in cancer cells. The persistent activation of STAT3 could be mediated by autocrine and/or paracrine cytokine loops through the JAK family, as well as activation of tyrosine kinases such as EGFR and SRC [29-32]. Persistent activation of STAT3 plays a critical role in tumor progression by promoting cell proliferation, cell survival, angiogenesis and tumor immune evasion, and is associated with a poor prognosis for ovarian cancer patients [29,33-35].

In this study, we investigated the mechanism for the limited activity of the EGFR inhibitor, gefitinib, in ovarian cancer cells, and found that increased activity of STAT3 after gefitinib treatment could partially explain the gefitinib resistance. Inhibition of STAT3 activity with a JAK inhibitor (JAKi) significantly enhanced the efficacy of gefitinib against human ovarian cancer cells both *in vitro* and in *vivo*. Our findings indicate that combined treatment with the EGFR inhibitor, gefitinib, and a JAK/STAT3 pathway inhibitor could potentially improve ovarian cancer treatment success.

## Results

### Effects of gefitinib on signaling pathways in human ovarian cancer cells

**Figure 1:**
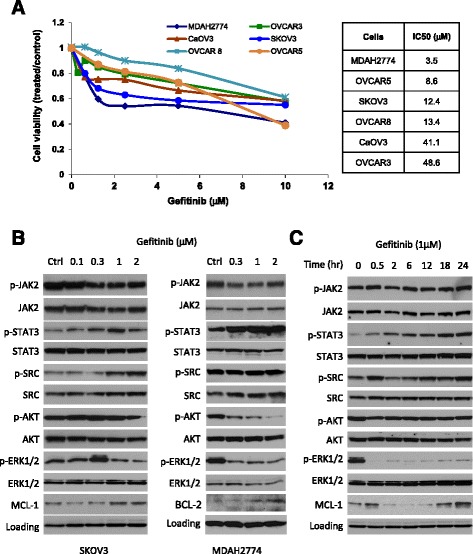
Anti-tumor activity of gefitinib in ovarian cancer cells. **(A)** Human ovarian cancer cell lines were treated with the indicated concentrations of gefitinib. Cell viability was determined 72 h later. The IC50 was determined by the Chou-Talalay method. **(B)** Western blots showing the dose-dependent effect of gefitinib on downstream signaling in human ovarian cancer cells. SKOV3 and MDAH2774 cells were incubated with increasing amounts of gefitinib for 24 h. **(C)** Western blots showing the time-dependent effect of gefitinib on downstream signaling in SKOV3 cells. SKOV3 cells were incubated with 1 μM gefitinib and collected at the indicated time points. GAPDH and actin were used as a loading control for SKOV3 and MDAH2774 cells, respectively. Results are representative of 2–5 preparations.

To study the anti-tumor activity of gefitinib in human ovarian cancer, we first tested the effects of gefitinib on the proliferation and viability of SKOV3 and other established human ovarian cancer cells. Cells were incubated with increasing concentrations of gefitinib and cell viability was determined 72 h later. As shown in Figure 1A, gefitinb inhibited cell viability with IC50s ranging from 3.5 μM to 49 μM. These IC50s are much higher than the IC50 for sensitive lung cancer cells [36].

It has been suggested that cancer cells often use alternative cell survival pathways to overcome growth inhibition induced by single drug treatment. In ovarian cancer cells, many survival pathways are persistently activated, including AKT, ERK, SRC and STAT3 signaling. To understand the effect of gefitinib on these signaling pathways in ovarian cancer cells, SKOV3 and MDAH2774 cells were incubated with increasing concentrations of gefitinib followed by Western blot analysis. Our results demonstrated that gefitinib significantly increased phosphorylation of STAT3, but not JAK2, in a dose dependent manner (Figure 1B). As a result of increased pSTAT3, the expression of MCL-1 and BCL-2, two STAT3 downstream genes, was also significantly increased in SKOV3 and MDAH2774, respectively. However, phosphorylation of ERK and AKT was decreased either in both cell lines (p-ERK) or only in MDAH2774 (p-AKT). Gefitinib-induced STAT3 phosphorylation occurred in a time- dependent manner in SKOV3 cells, reaching a peak at 18 h (Figure 1C). These results suggest that the potential efficacy of gefitinib in human ovarian cancer cells might be attenuated by activation of the JAK/STAT3 survival pathway.

### Cell viability after combined treatment with gefitinib and a small molecule inhibitor of the JAK/STAT3 pathway

**Figure 2:**
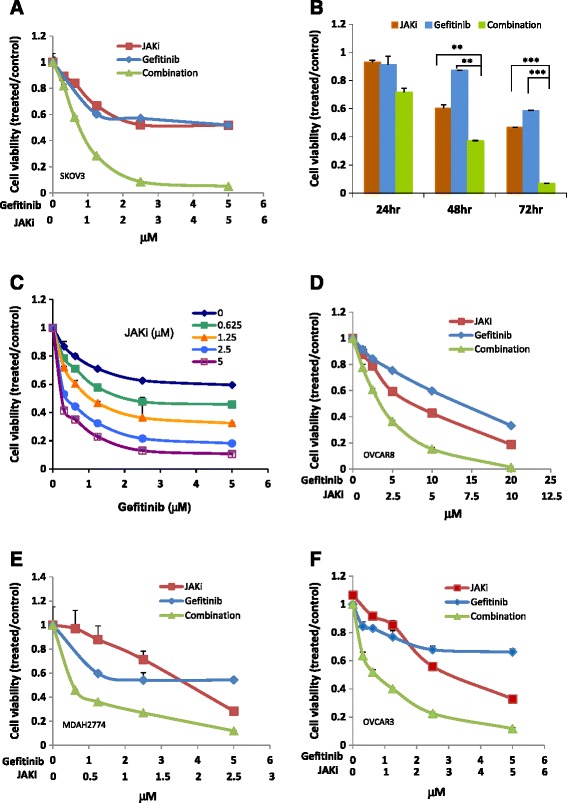
Suppressing the JAK/STAT3 pathway with a small molecule inhibitor of JAK (JAKi) enhanced the anti-tumor activity of gefitinib in human ovarian cancer cells. **(A)** SKOV3 cells were treated with JAKi (AZD1480) or gefitinib alone or in combination at various concentrations in a fixed molar ratio 1:1. Cell viability was determined 72 h later. **(B)** SKOV3 cells were treated with JAKi (5 μM) or gefitinb (5 μM) either as single agent or in combination. Cell viability was determined 24 h, 48 h and 72 h later. **(C)** SKOV3 cells were incubated with increasing amounts of gefitinib in the presence of a fixed concentration of JAKi. Cell viability was determined 72 h later. OVCAR8 **(D)**, MDAH2774 **(E)** and OVCAR 3 cells **(F)** were treated with gefitinib or JAKi either alone or in combination. Cell viability was determined 72 h later. Results are representative of 2–5 preparations.

To understand whether inhibiting JAK/STAT3 pathway could increase the sensitivity of human ovarian cancer cells to gefitinib, a recently identified JAKi, AZD1480, was tested for its effect on cell viability when combined with gefitinib. This JAKi has been shown to inhibit phosphorylation of STAT3 in various cancer cells, including human ovarian cancer cell lines MDAH2774 and SKOV3 [37-40]. We treated SKOV3 ovarian cancer cells with gefitinib either alone or in combination with JAKi. The combination treatment decreased cell viability much more robustly than either agent alone (Figure 2A). This effect was time-dependent with the strongest cytotoxicity seen at 72 h (Figure 2B). When gefitinib was combined with JAKi in a fixed 1:1 molar ratio, the concentration of gefitinib that gave 50% and 75% inhibition in SKOV3 cells decreased by about 16 fold from 12 μM to 0.74 μM and 117 fold from 164 μM to 1.4 μM, respectively (Figure 1 and Tables 1 and 2). When fixed concentrations of JAKi were incubated with increasing concentrations of the EGFR inhibitor gefitinib, highest anti-cancer activity was seen in the highest combined doses (Figure 2C).

**Table 1:** **Synergistic interaction between JAKi and gefitinib in variety of molar ratios on the viability of SKOV3 cells**

**JAKi: Gefitinib**	**Combination index (CI)**			**IC50 (μ** **M)**	
	**ED50**	**ED75**	**ED90**	**JAKi**	**Gefitinib**
**1:3**	0.7	0.14	0.052	0.87	2.61
**1:2**	0.34	0.092	0.044	0.55	1.09
**1:1**	0.32	0.11	0.048	0.74	0.74
**2:1**	0.35	0.13	0.061	1.01	0.55
**4:1**	0.35	0.16	0.08	1.15	0.29
**8:1**	0.48	0.26	0.15	1.71	0.21

**Table 2:** **Synergistic interaction between JAKi and gefitinib on cell viability in a variety of human ovarian cancer cell lines**

**Cells**	**JAKi: Gefitinib**	**Combination index (CI)**			**Fold reduction (gefitinib)**		
		**ED50**	**ED75**	**ED90**	**IC50**	**IC75**	**IC90**
**SKOV3**	1:1	0.32	0.11	0.048	16.13	117.14	1764.86
**MDAH2774**	1:2	0.36	0.41	0.59	5.33	10.92	22.38
**OVCAR 3**	1:4	0.29	0.29	0.37	33.11	145.78	645.24
**OVCAR 8**	1:2	0.69	0.57	0.47	4.15	6.52	10.23

To understand whether the increased activity was additive or synergistic, we determined the combination index (CI) according to the Chou-Talalay method (CI = 1, additive effect; CI < 1, synergism; CI > 1 antagonism) [41]. SKOV3 cells were incubated with the combination of JAKi (fixed at 5 μM) and gefitinib (0.625 to 30 μM) at various JAKi/gefitinib molar ratios (8:1, 4:1, 2:1, 1:1, 1:2 and 1:3). As shown in Table 1, the combination treatment produced a very strong synergism at each molar ratio. But it appears the combination at 1:1 molar ratio produced stronger synergy and a lower IC50 for both agents in the SKOV3 cells. We next investigated whether combination treatment with gefitinib and JAKi was also more effective than either agent alone in other human ovarian cancer cells. As shown in Figure 2D-F and Table 2, mild to very strong synergy was also observed in other ovarian cell lines, including OVCAR 8, MDAH2774, and OVCAR3.

Our results thus far indicate that STAT3 inhibition can enhance the sensitivity of ovarian cancer cells to gefitinib. We next determined whether inhibition of other pathways such as the AKT/mTOR, MEK/ERK or SRC pathway would have a similar effect. As shown in Table 3, inhibition of SRC with saracatinib or MEK with AZD6244 was not able to effectively reduce the IC50 of gefitinib in SKOV3 cells. An AKT inhibitor, MK2206, or an mTOR inhibitor, RAD001, synergistically increased gefitinib sensitivity in the ovarian cells, however, was less effective than JAKi. The concentrations of gefitinib required to induce 75% and 90% inhibition were decreased much more robustly in the presence of JAKi compared with the other inhibitors. These results suggest that inhibiting the JAK/STAT3 pathway was more effective than inhibiting other signaling pathways in sensitizing these human ovarian cancer cells to gefitinib.

**Table 3:** **Effects of combining gefitinib with JAK inhibitor (AZD1480), SRC inhibitor (saracatinib), MEK inhibitor (AZD6244), AKT inhibitor (MK2204) and mTOR inhibitor (RAD001), on the viability of SKOV3 cells**

**Inhibitor**	**Inhibitor: Gefitinib**	**Combination index (CI)**			**Fold reduction (gefitinib)**		
		**ED50**	**ED75**	**ED90**	**IC50**	**IC75**	**IC90**
**JAK**	1:1	0.32	0.11	0.048	16.13	117.14	1764.86
**SRC**	1:3	0.66	0.27	0.11	1.53	3.76	9.28
**MEK**	1:4	1.09	0.83	0.62	0.95	1.26	1.68
**AKT**	1:2	0.32	0.35	0.59	6.92	3.51	1.77
**mTOR**	1:1	0.1	0.22	0.48	10.16	4.6	2.06

### Effects of combination treatment with gefitinib and JAKi on apoptosis

**Figure 3:**
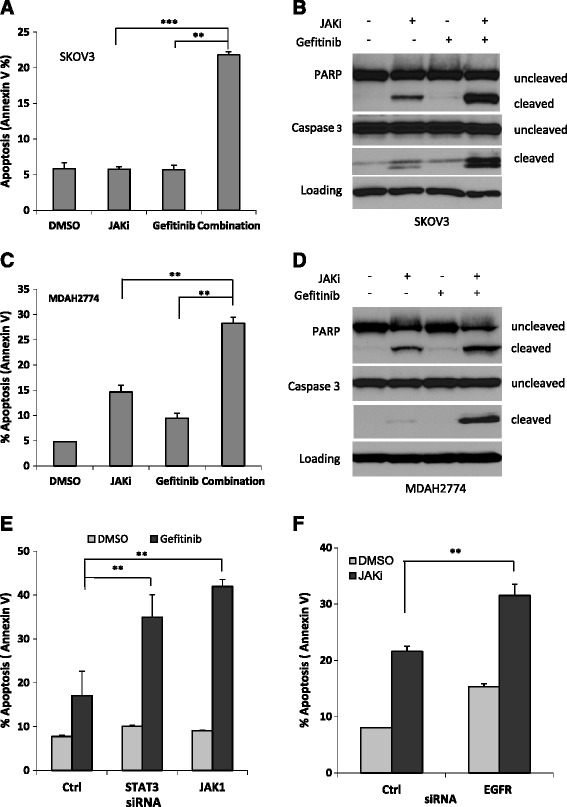
Suppressing JAK/STAT3 signaling enhanced gefitinib-induced apoptosis in human ovarian cancer cells. SKOV3 **(A&B)** and MDAH2774 **(C&D)** cells were treated with gefitinib, JAKi, either alone or together, for 48 h. Apoptosis was determined by flow cytometry using Annexin V and PI staining **(A&C)** or for cleaved poly-ADP ribose polymerase (PARP) and cleaved caspase-3 by Western blot **(B&D)**. **, *P <* 0.005; ***, P < 0.005, combination *vs.* JAKi alone or gefitinib alone. **(E)** SKOV3 cells were transfected with siRNA against STAT3, JAK1, or control siRNA and treated with gefitinib or **(F)** transfected with siRNA against EGFR and treated with JAKi. After 48 h, cells were harvested and evaluated for apoptosis using Annexin V staining. *, *P <* 0.05; **, *P <* 0.005, *vs.* control siRNA.

Next, we determined whether the synergistic effects of JAKi and gefitinib extended to induction of apoptosis. Cells were treated with gefitinib and JAKi either alone or in combination for 48 h and the number of apoptotic cells was determined by Annexin V staining. As shown in Figure 3, gefitinib-induced apoptosis increased from 5.7% to 21.8% in SKOV3 cells and from 9.5% to 28.2% in MDAH2774 cells when combined with JAKi (Figures 3A and 3C). Consistent with the Annexin V staining results, more cleaved caspase 3 and cleaved poly-ADP ribose polymerase (PARP) were generated in the cells that were treated with both gefitinib and JAKi (Figures 3B and 3D). These results indicate that inhibition of the JAK/STAT3 pathway could effectively enhance the sensitivity of these human ovarian cancer cells to gefitinib by promoting apoptosis.

### Effect of gefitinib on the viability of cells with JAK/STAT3 knockdown

To further understand whether inhibition of the JAK/STAT3 pathway could increase the sensitivity of human ovarian cancer cells to gefitinib, we tested whether gefitinib sensitivity could be enhanced by siRNA-mediated knockdown of JAK or STAT3. Our previous studies have shown that depletion of JAK1, but not JAK2, abolished phosphorylation of STAT3 in SKOV3 and MDAH2774 cells, suggesting that JAK1 is a major kinase responsible for STAT3 phosphorylation in these two cell lines. We here examined the sensitivity of ovarian cancer cells to gefitinib when the JAK/STAT3 pathway was depleted either with JAK1 siRNA or STAT3 siRNA. In response to gefitinib treatment, the number of apoptotic cells significantly increased from 17% in cells transfected with a control siRNA to 35% or 41% in cells transfected with siRNA against JAK1 or STAT3, respectively (Figure 3E). This result further demonstrates that inhibition of JAK/STAT3 pathway is an effective way to enhance gefitinib activity in ovarian cancer.

Similarly, we also investigated whether knockdown of EGFR expression could increase JAKi-induced apoptosis. As shown in Figure 3F, JAKi-induced apoptosis increased from 21% to 36% when cells were transfected with EGFR siRNA, suggesting that inhibition of EGFR potentiate the inhibitory activity of JAKi in ovarian cancer.

### Effects of combined gefitinib and JAKi treatment on downstream signaling pathways

**Figure 4:**
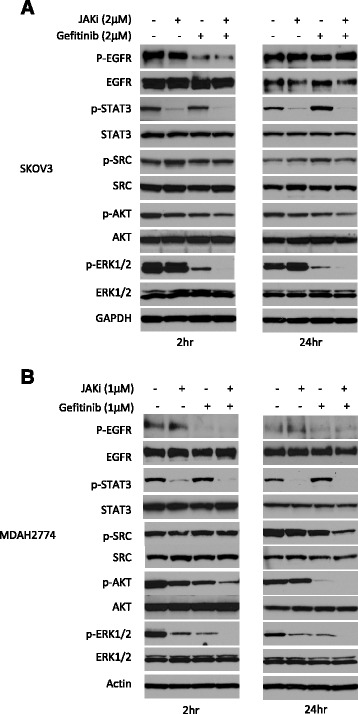
Western blots show that combination treatment with JAKi and gefitinib caused attenuation of multiple signaling pathways. SKOV3 **(A)** and MDAH2774 **(B)** cells were treated with JAKi, gefitinib or the combination for 2 h and 24 h. Results are representative of 2–4 preparations.

To further investigate the synergistic interaction between gefitinib and JAKi, we evaluated the molecular changes in ovarian cancer cells after treatment with gefitinib and JAKi either alone or in combination. As shown in Figure 4, treatment with gefitinib alone resulted in a decreased level of p-ERK1/2, but not p-STAT3 in both cell lines at 2 h and 24 h. Treatment with JAKi alone inhibited p-STAT3 as expected in both cell lines. The combined treatment with both gefitinib and JAKi led to inhibition of p-STAT3 (Figure 4), as well as STAT3 downstream genes, MCL-1 and BCL-2 (data not shown). The inhibition of p-ERK caused by combined treatment was considerably greater compared to any single treatment in both cell lines. The dual treatment also caused increased inhibition of p-AKT in both cell lines, although the inhibition was not very strong in SKOV3 cells. A stronger reduction of p-SRC by combination treatment was also found in MDAH2774 cells. Taken together, these results demonstrate that combined targeting of both, EGFR and JAK/STAT3 pathways, can inhibit multiple survival pathways and results in greater inhibition of p-ERK.

### Effect of combination treatment on ovarian cancer growth in mice

**Figure 5:**
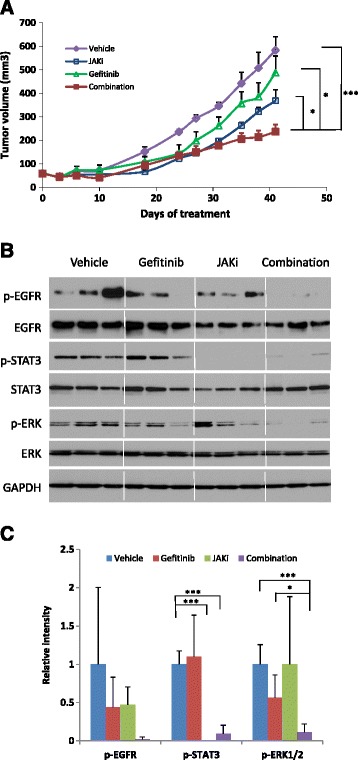
JAKi increased the anti-tumor activity of gefitinib in mice. **(A)** SKOV3 cells were implanted subcutaneously into the right flank of nude mice. Tumors were treated with vehicle, JAKi (30 mg/kg), gefitinib (150 mg/kg) or their combination by oral gavage once daily. Tumor growth was measured twice a week. Data represents means ± SD (n = 8-10). *, *P <* 0.05; ***, *P <* 0.005, combination *vs.* vehicle, or gefitinib alone or JAKi alone. **(B)** Western blots show the effects of treatment on the indicated intracellular signaling pathways in tumor tissue. **(C)** Relative levels of p-EGFR, p-STAT3, p-ERK were determined by measuring the density of each band and normalized to that of GAPDH. Densitometry data were relative changes in protein expression and were mean ± SD of 2–3 preparations. *, *P <* 0.05; ***, *P <* 0.0005.

Next we investigated whether the combination treatment could suppress tumor growth *in vivo* more effectively than either treatment alone. NSG mice were subcutaneously inoculated with SKOV3 ovarian cancer cells. When the tumors were palpable, mice were randomized into four groups and treated with vehicle control, gefitinib, JAKi or gefitinib plus JAKi through oral gavage. No toxicity was observed in mice with any of the treatments, whether the drugs were used alone or in combination, as indicated by absence of significant (>5%) change in body weight (not shown). Treatment with gefitinib alone was not very effective; the tumor volume was similar to the vehicle-treated group (Figure 5A). Treatment with JAKi alone at a daily dose of 30 mg/kg decreased the tumor burden by 37%. However, the combination treatment further decreased the tumor volume by another 22% (Figure 5A), suggesting that the combination treatment was more effective than any single treatment.

To investigate molecular changes in the tumors upon treatment, tumor tissue lysates were analyzed for the expression of p-EGFR, p-STAT3 and p-ERK by Western blot analysis. As shown in Figure 5B and 5C, phosphorylation of STAT3 was blocked in the presence of JAKi either alone or in combination with gefitinib. Combination of gefitinib with JAKi treatment led to greater inhibition of p-ERK, which was consistent with *in vitro* results shown in Figure 4. Overall, these results support a consistent synergistic effect of EGFR and JAK/STAT3 inhibition on ovarian cancer cell growth and survival both *in vitro* and *in vivo* and a potential role for multiple survival pathways in this effect.

## Discussion

To improve the clinical benefit of targeting EGFR in the treatment of ovarian cancer, we investigated the mechanism of resistance to EGFR inhibition. We demonstrate, for the first time, that inhibition of EGFR significantly increased phosphorylation of STAT3 in ovarian cancer cells and blocking STAT3 activation led to enhanced anti-tumor activity of gefitinib both *in vitro* and *in vivo*.

Studies in non-small cell lung cancer and other cancers have found that resistance to anti-EGFR agents is common and occurs through several proposed mechanisms, such as *de novo* resistance due to genetic alteration of receptors or downstream signaling molecules and acquired resistance due to activation of alternative signaling pathways [25-27,36]. However, little is known about the relevance of these mechanisms to ovarian cancer. Unlike non-small cell lung cancer, mutations in ovarian cancer are rare [23,42,43]. The concurrent activation of multiple signaling pathways, including EGFR, PI3/AKT, MEK/ERK and JAK/STAT3, appears to be more common in ovarian cancer and raises an important question about the involvement of these signaling pathways in the development of drug resistance [4]. Targeting the PI3K/AKT/mTOR pathway has been shown to increase anti-tumor activity of inhibiting EGFR [44,45]. In the present study, we found that inhibition of EGFR in ovarian cancer cells increased phosphorylation of STAT3 in both SKOV3 and MDAH2774 cells in a time- and dose-dependent manner. Blockade of STAT3 pathway synergistically increased anti-tumor activity of gefitinib, as well as another EGFR inhibitor (erlotinib) (data not shown). This finding is consistent with previous reports in some other tumors, including glioma, lung and pancreatic cancer [46-49]. Blocking other survival pathways, such as AKT, mTOR, ERK and SRC, also enhanced the sensitivity to gefitinib in our study, however, inhibition of these pathways was not as effective as inhibiting JAK. Relative to the other pathways considered in this study, targeting STAT3 appeared to be the most effective way to increase the therapeutic efficacy of anti-EGFR therapy in these ovarian cancer cells. Taken together, these results provide a molecular mechanism underlying resistance to EGFR inhibition in human ovarian cancer.

The gefitinib-induced pSTAT3 was abolished in the presence of JAK inhibitor, implying JAK might be involved in the activation of STAT3. Our previous study has demonstrated that the constitutive phosphorylation of STAT3 is largely mediated through JAK1 in these ovarian cancer cells, suggesting a possibility of JAK1 as a main kinase responsible for activation of STAT3 by EGF blockade [40]. Since pJAK1 was not detectable by Western blot using currently commercial available antibody (data not shown) in these cell lines, it remains to be addressed whether gefitinib also induce phosphorylation and activation of JAK1. The gefitinib–induced activation of STAT3 is unlikely a temporal activation as STAT3 remained to be activated through whole treatment. Unlike cytokine-induced phosphorylation of STAT3, which occurs at early time, the gefitinib-induced phosphorylation of STAT3 appeared to be stronger at later points, suggesting that JAK/STAT3 might be activated by gefitinib via an indirect pathway. Further study is warranted to define the molecular mechanism underlying the activation of STAT3 by gefitinib.

While inhibiting a single pathway including EGFR and JAK/STAT3 was not sufficient to significantly block cell growth and survival (Figures 1 and 2), dual blockade of STAT3 and EGFR resulted in simultaneous attenuations of multiple survival pathways and dramatically increased anti-tumor activity *in vitro* and *in vivo*. Therefore, concurrent activation of multiple signaling pathways might be the critical force that drives ovarian cancer cells to proliferate and survive. To achieve a maximum anti-tumor activity, simultaneous blockade of multiple survival pathways may be required. EGFR is one of the main kinases involved in activation of a number of downstream survival pathways, such as ERK, AKT and SRC, in ovarian cancer cells. Inhibition of EGFR could effectively increase anti-tumor activity of agents targeting other signaling pathways, such as STAT3 or AKT. Therefore, blocking the EGFR pathway could be an important strategy to improve the impact of targeted therapy in ovarian cancer.

While activating EGFR mutations have been known as predictors for response to anti-EGFR therapy in lung cancer, STAT3 activation was recently suggested as a potential predictive marker for resistance to anti-EGFR therapies in patients with mCRC and NSCLC, given the correlation between the activation of STAT3 and resistance of cells to anti-EGFR therapy [50,51]. Further studies are required to address whether p-STAT3 could be a potential biomarker to predict the response to gefitinib in ovarian cancer.

## Conclusions

In this study, our results, for the first time, demonstrate that feedback activation of STAT3 might explain in part why gefitinib is not effective against human ovarian cancer. Combining EGFR blockade with suppression of JAK/STAT3 signaling is more effective in inhibiting ovarian cancer growth than inhibition of either pathway alone. This study may provide a potential combination therapeutic strategy for the treatment of advanced ovarian cancer.

## Materials and methods

### Reagents

JAKi, AZD1480, was kindly provided by AstraZeneca. Antibodies against p-STAT3 (Y705), STAT3, p-ERK (T202/Y204) , ERK, p-AKT (S473), p-SRC (Y416), SRC, p-EGFR (Y1068), EGFR, p-JAK2 (Y1007/1008), JAK2, BCL-2, PARP, Caspase 3 and GAPDH were obtained from Cell Signaling Technology (Danvers, MA). The antibodies against AKT and MCL-1 were from Santa Cruz Biotechnology (Dallas, TX). The antibody against actin was from Sigma (St. Louis, MO).

### Cell viability assays

Cells (4000 per well for most cells, 7000 per well for MDAH2774) were plated in 96-well plate format in 100 μl growth medium. Cells were treated with DMSO or drugs the next day at the indicated concentrations and incubated for an additional 1 to 3 days. Viable cells were determined either by the MTS assay (Promega, Madison, WI, USA) or the acid phosphatase assay [52]. For the MTS assay, 25 μl MTS solution was added directly into each well according to the manufacturer’s instructions. For the acid phophatase assay, all the media was removed and p-nitrophenyl phosphate substrate (10 mM 100 μl) was added into each well and incubated at 37°C for 45mins. NaOH was added to stop the reaction and the absorbance was read at 415 nM. The IC50 was determined using the Calcusyn software (Biosoft, Ferguson, MO).

### Determination of combination index (CI)

The combination index (CI) was determined using the Chou-Talalay method [41] using the Calcusyn software (Biosoft, MO).

### Annexin V staining

Apoptosis was measured using the Annexin V apoptosis detection kit (BD bioscience). Briefly, ovarian cancer cells were treated with gefitinib, JAKi or both compounds. After 48 h, both floating and attached cells were collected and stained with FITC-Annexin V and PI (propidium iodide). The staining intensity was then quantified using fluorescence-activated cell sorting (FACS).

### Western blot analysis

Western blots were performed as described previously [53]. Cells were grown in complete medium overnight and treated with DMSO or drugs at various concentrations for various times. Cells were washed in cold PBS and lysed in RIPA lysis buffer (Thermo Scientific) containing Halt protease and phosphatase inhibitors (Thermo Scientific). Proteins were quantified using BCA protein assay reagent (Thermo Scientific). Equal amounts of protein were separated by SDS-polyacrylamide gel electrophoresis, transferred to polyvinylidene fluoride membranes and incubated with total and phosphorylated protein-specific antibodies. Binding of the primary antibody was detected using a horseradish peroxidase (HRP)-conjugated secondary antibody and chemiluminescent substrates (Thermo Scientific).

### Transfection with small interfering RNA (siRNA)

RNAiMAX (Invitrogen) was used to transfect siRNA, according to the manufacturer’s instructions. After 24 h of incubation with RNAi (Santa Cruz Biotechnology), cells were treated with drugs as shown.

### Animal models

All animal studies were carried out under protocols approved by the Institutional Animal Care and Use Committee (IACUC) at City of Hope in accordance with all applicable federal, state, and local requirements and institutional guidelines. SKOV3 cells (5×10^6^ in 100 μl) were inoculated subcutaneously into the right flank of 6- to 8- week-old female NOD/SCID/IL2Rgamma null (NSG) mice. Once the tumors were palpable, animals were randomized into groups of 10 to achieve an equal distribution of tumor sizes in all treatment groups. Mice were then treated by oral gavage daily with vehicle, JAKi (30 mg/kg), gefitinib (150 mg/kg), or a combination of both agents. The doses for these two drugs were chosen based on previously published studies [37-39,54]. Tumor volumes were assessed using calipers twice a week. Tumor volumes were determined using the formula (Width)^2^ × Length × 0.52. Body weight was monitored weekly as an indicator of drug-induced toxicity and overall health of the mice.

### Statistical analysis

Data are presented as mean ± S.D. Student’s t-test was used to compare the means of two groups. All the experiments were repeated 2 to 4 times. P < 0.05 was considered statistically significant.

## References

[1] Vaughan S, Coward JI, Bast RC, Berchuck A, Berek JS, Brenton JD (2011). Rethinking ovarian cancer: recommendations for improving outcomes. Nat Rev Cancer.

[2] Cannistra SA (2004). Cancer of the ovary. N Engl J Med.

[3] Cristea M, Han E, Salmon L, Morgan RJ (2010). Practical considerations in ovarian cancer chemotherapy. Ther Adv Med Oncol.

[4] Yap TA, Carden CP, Kaye SB (2009). Beyond chemotherapy: targeted therapies in ovarian cancer. Nat Rev Cancer.

[5] Bast RC, Hennessy B, Mills GB (2009). The biology of ovarian cancer: new opportunities for translation. Nat Rev Cancer.

[6] Campos SM, Ghosh S (2010). A current review of targeted therapeutics for ovarian cancer. J Oncol.

[7] Lemmon MA, Schlessinger J (2010). Cell Signaling by Receptor Tyrosine Kinases. Cell.

[8] Citri A, Yarden Y (2006). EGF–ERBB signalling: towards the systems level. Nat Rev Mol Cell Biol.

[9] Scaltriti M, Baselga J (2006). The epidermal growth factor receptor pathway: a model for targeted therapy. Clin Cancer Res.

[10] Yarden Y, Sliwkowski MX (2001). Untangling the ErbB signalling network. Nat Rev Mol Cell Biol.

[11] Yarden Y, Pines G (2012). The ERBB network: at last, cancer therapy meets systems biology. Nat Rev Cancer.

[12] Ciardiello F, Tortora G (2008). EGFR antagonists in cancer treatment. N Engl J Med.

[13] Cataldo VD, Gibbons DL, Perez-Soler R, Quintas-Cardama A (2011). Treatment of non-small-cell lung cancer with erlotinib or gefitinib. N Engl J Med.

[14] Maemondo M, Inoue A, Kobayashi K, Sugawara S, Oizumi S, Isobe H (2010). Gefitinib or chemotherapy for non-small-cell lung cancer with mutated EGFR. N Engl J Med.

[15] Psyrri A, Kassar M, Yu Z, Bamias A, Weinberger PM, Markakis S (2005). Effect of epidermal growth factor receptor expression level on survival in patients with epithelial ovarian cancer. Clin Cancer Res.

[16] Siwak DR, Carey M, Hennessy BT, Nguyen CT, McGahren Murray MJ, Nolden L (2010). Targeting the epidermal growth factor receptor in epithelial ovarian cancer: current knowledge and future challenges. J Oncol.

[17] Bartlett JM, Langdon SP, Simpson BJ, Stewart M, Katsaros D, Sismondi P (1996). The prognostic value of epidermal growth factor receptor mRNA expression in primary ovarian cancer. Br J Cancer.

[18] Fischer-Colbrie J, Witt A, Heinzl H, Speiser P, Czerwenka K, Sevelda P (1997). EGFR and steroid receptors in ovarian carcinoma: comparison with prognostic parameters and outcome of patients. Anticancer Res.

[19] Niikura H, Sasano H, Sato S, Yajima A (1997). Expression of epidermal growth factor-related proteins and epidermal growth factor receptor in common epithelial ovarian tumors. Int J Gynecol Pathol.

[20] Murphy M, Stordal B (2011). Erlotinib or gefitinib for the treatment of relapsed platinum pretreated non-small cell lung cancer and ovarian cancer: a systematic review. Drug Resist Updat.

[21] Blank SV, Christos P, Curtin JP, Goldman N, Runowicz CD, Sparano JA (2010). Erlotinib added to carboplatin and paclitaxel as first-line treatment of ovarian cancer: A phase II study based on surgical reassessment. Gynecol Oncol.

[22] Sheng Q, Liu J (2011). The therapeutic potential of targeting the EGFR family in epithelial ovarian cancer. Br J Cancer.

[23] Schilder RJ, Sill MW, Chen X, Darcy KM, Decesare SL, Lewandowski G (2005). Phase II study of gefitinib in patients with relapsed or persistent ovarian or primary peritoneal carcinoma and evaluation of epidermal growth factor receptor mutations and immunohistochemical expression: a Gynecologic Oncology Group Study. Clin Cancer Res.

[24] Gordon AN, Finkler N, Edwards RP, Garcia AA, Crozier M, Irwin DH (2005). Efficacy and safety of erlotinib HCl, an epidermal growth factor receptor (HER1/EGFR) tyrosine kinase inhibitor, in patients with advanced ovarian carcinoma: results from a phase II multicenter study. Int J Gynecol Cancer.

[25] Chong CR, Janne PA (2013). The quest to overcome resistance to EGFR-targeted therapies in cancer. Nat Med.

[26] Camidge DR, Pao W, Sequist LV (2014). Acquired resistance to TKIs in solid tumours: learning from lung cancer. Nat Rev Clin Oncol.

[27] Niederst MJ, Engelman JA (2013). Bypass Mechanisms of Resistance to Receptor Tyrosine Kinase Inhibition in Lung Cancer. Sci Signal.

[28] Darnell JE, Kerr IM, Stark GR (1994). Jak-STAT pathways and transcriptional activation in response to IFNs and other extracellular signaling proteins. Science.

[29] Yu H, Pardoll D, Jove R (2009). STATs in cancer inflammation and immunity: a leading role for STAT3. Nat Rev Cancer.

[30] Bromberg JF, Wrzeszczynska MH, Devgan G, Zhao Y, Pestell RG, Albanese C (1999). Stat3 as an oncogene. Cell.

[31] Bromberg J, Wang TC (2009). Inflammation and cancer: IL-6 and STAT3 complete the link. Cancer Cell.

[32] Catlett-Falcone R, Landowski TH, Oshiro MM, Turkson J, Levitzki A, Savino R (1999). Constitutive activation of Stat3 signaling confers resistance to apoptosis in human U266 myeloma cells. Immunity.

[33] Rosen DG, Mercado-Uribe I, Yang G, Bast RC, Amin HM, Lai R (2006). The role of constitutively active signal transducer and activator of transcription 3 in ovarian tumorigenesis and prognosis. Cancer.

[34] Tempfer C, Zeisler H, Sliutz G, Haeusler G, Hanzal E, Kainz C (1997). Serum evaluation of interleukin 6 in ovarian cancer patients. Gynecol Oncol.

[35] Lane D, Matte I, Rancourt C, Piche A (2011). Prognostic significance of IL-6 and IL-8 ascites levels in ovarian cancer patients. BMC Cancer.

[36] Guix M, Faber AC, Wang SE, Olivares MG, Song Y, Qu S (2008). Acquired resistance to EGFR tyrosine kinase inhibitors in cancer cells is mediated by loss of IGF-binding proteins. J Clin Invest.

[37] Hedvat M, Huszar D, Herrmann A, Gozgit JM, Schroeder A, Sheehy A (2009). The JAK2 inhibitor AZD1480 potently blocks Stat3 signaling and oncogenesis in solid tumors. Cancer Cell.

[38] Scuto A, Krejci P, Popplewell L, Wu J, Wang Y, Kujawski M (2011). The novel JAK inhibitor AZD1480 blocks STAT3 and FGFR3 signaling, resulting in suppression of human myeloma cell growth and survival. Leukemia.

[39] Xin H, Herrmann A, Reckamp K, Zhang W, Pal S, Hedvat M (2011). Antiangiogenic and antimetastatic activity of JAK inhibitor AZD1480. Cancer Res.

[40] Wen W, Liang W, Wu J, Kowolik CM, Buettner R, Scuto A (2014). Targeting JAK1/STAT3 signaling suppresses tumor progression and metastasis in a peritoneal model of human ovarian cancer. Mol Cancer Ther.

[41] Chou TC (2010). Drug combination studies and their synergy quantification using the Chou-Talalay method. Cancer Res.

[42] Lacroix L, Pautier P, Duvillard P, Motte N, Saulnier P, Bidart JM (2006). Response of ovarian carcinomas to gefitinib-carboplatin-paclitaxel combination is not associated with EGFR kinase domain somatic mutations. Int J Cancer.

[43] Steffensen KD, Waldstrom M, Olsen DA, Corydon T, Lorentzen KA, Knudsen HJ (2008). Mutant epidermal growth factor receptor in benign, borderline, and malignant ovarian tumors. Clin Cancer Res.

[44] Glaysher S, Bolton LM, Johnson P, Atkey N, Dyson M, Torrance C (2013). Targeting EGFR and PI3K pathways in ovarian cancer. Br J Cancer.

[45] Muranen T, Selfors LM, Worster DT, Iwanicki MP, Song L, Morales FC (2012). Inhibition of PI3K/mTOR leads to adaptive resistance in matrix-attached cancer cells. Cancer Cell.

[46] Dowlati A, Nethery D, Kern JA (2004). Combined inhibition of epidermal growth factor receptor and JAK/STAT pathways results in greater growth inhibition in vitro than single agent therapy. Mol Cancer Ther.

[47] Lo HW, Cao X, Zhu H, Ali-Osman F (2008). Constitutively activated STAT3 frequently coexpresses with epidermal growth factor receptor in high-grade gliomas and targeting STAT3 sensitizes them to Iressa and alkylators. Clin Cancer Res.

[48] Jaganathan S, Yue P, Turkson J (2010). Enhanced sensitivity of pancreatic cancer cells to concurrent inhibition of aberrant signal transducer and activator of transcription 3 and epidermal growth factor receptor or Src. J Pharmacol Exp Ther.

[49] Sen M, Joyce S, Panahandeh M, Li C, Thomas SM, Maxwell J (2012). Targeting Stat3 Abrogates EGFR Inhibitor Resistance in Cancer. Clin Cancer Res.

[50] Dobi E, Monnien F, Kim S, Ivanaj A, N'Guyen T, Demarchi M (2013). Impact of STAT3 phosphorylation on the clinical effectiveness of anti-EGFR-based therapy in patients with metastatic colorectal cancer. Clin Colorectal Cancer.

[51] Haura EB, Sommers E, Song L, Chiappori A, Becker A (2010). A pilot study of preoperative gefitinib for early-stage lung cancer to assess intratumor drug concentration and pathways mediating primary resistance. J Thorac Oncol.

[52] Yang TT, Sinai P, Kain SR (1996). An acid phosphatase assay for quantifying the growth of adherent and nonadherent cells. Anal Biochem.

[53] Lu J, Zhang K, Nam S, Anderson RA, Jove R, Wen W (2010). Novel angiogenesis inhibitory activity in cinnamon extract blocks VEGFR2 kinase and downstream signaling. Carcinogenesis.

[54] Sirotnak FM, Zakowski MF, Miller VA, Scher HI, Kris MG (2000). Efficacy of cytotoxic agents against human tumor xenografts is markedly enhanced by coadministration of ZD1839 (Iressa), an inhibitor of EGFR tyrosine kinase. Clin Cancer Res.

